# Detection of alpha‐synuclein conformational variants from gastro‐intestinal biopsy tissue as a potential biomarker for Parkinson's disease

**DOI:** 10.1111/nan.12486

**Published:** 2018-05-08

**Authors:** C. Ruffmann, N. Bengoa‐Vergniory, I. Poggiolini, D. Ritchie, M. T. Hu, J. Alegre‐Abarrategui, L. Parkkinen

**Affiliations:** ^1^ Oxford Parkinson's Disease Centre Nuffield Department of Clinical Neurosciences University of Oxford John Radcliffe Hospital Oxford UK; ^2^ Oxford Parkinson's Disease Centre Department of Physiology, Anatomy and Genetics University of Oxford Oxford UK; ^3^ National CJD Research & Surveillance Unit Centre for Clinical Brain Sciences Deanery of Clinical Medicine University of Edinburgh Edinburgh UK

**Keywords:** Alpha‐synuclein, Biomarker, Gastro‐intestinal tract, Immunohistochemistry, Parkinson's disease, Prodromal

## Abstract

**Aims:**

Gastrointestinal (GI) α‐synuclein (aSyn) detection as a potential biomarker of Parkinson's disease (PD) is challenged by conflicting results of recent studies. To increase sensitivity and specificity, we applied three techniques to detect different conformations of aSyn in GI biopsies obtained from a longitudinal, clinically well‐characterized cohort of PD patients and healthy controls (HC).

**Methods:**

With immunohistochemistry (IHC), we used antibodies reactive for total, phosphorylated and oligomeric aSyn; with aSyn proximity ligation assay (AS‐PLA), we targeted oligomeric aSyn species specifically; and with paraffin‐embedded tissue blot (AS‐PET‐blot) we aimed to detect fibrillary, synaptic aSyn.

**Results:**

A total of 163 tissue blocks were collected from 51 PD patients (113 blocks) and 21 HC (50 blocks). In 31 PD patients, biopsies were taken before the PD diagnosis (Prodromal); while in 20 PD patients biopsies were obtained after diagnosis (Manifest). The majority of tissues blocks were from large intestine (62%), followed by small intestine (21%), stomach (10%) and oesophagus (7%). With IHC, four staining patterns were detected (*neuritic, ganglionic, epithelial and cellular*), while two distinct staining patterns were detected both with AS‐PLA (*cellular* and *diffuse* signal) and with AS‐PET‐blot (*aSyn‐localized* and *pericrypt* signal). The level of agreement between different techniques was low and no single technique or staining pattern reliably distinguished PD patients (Prodromal or Manifest) from HC.

**Conclusions:**

Our study suggests that detection of aSyn conformational variants currently considered pathological is not adequate for the diagnosis or prediction of PD. Future studies utilizing novel ultrasensitive amyloid aggregation assays may increase sensitivity and specificity.

## Introduction

Parkinson's disease (PD) is the second most prevalent neurodegenerative disease and a major cause of disability and socio‐economic burden. Currently, the diagnosis of PD rests on clinical criteria and can only be confirmed with a postmortem examination of the brain, which reveals intraneuronal inclusions of misfolded α‐synuclein (aSyn) protein as Lewy pathology coupled with neuronal loss in the substantia nigra (SN) 1. *In vivo* diagnostic biomarkers of PD are currently lacking and could help to identify individuals at a preclinical stage, possibly preceding significant neuronal loss in the SN. PD biomarkers would potentially allow us to identify subjects at risk, monitor disease progression, optimize patient inclusion into clinical trials and help us to assess the efficacy of future therapies 2.

Accumulation of aSyn is not limited to the central nervous system (CNS) and we have recently reviewed a growing body of literature showing that aSyn aggregation can be detected particularly in the enteric nervous system (ENS) in *postmortem* but also in readily and safely accessible *in vivo* biopsies of the gastro‐intestinal (GI) tract 3. Some studies suggest that aSyn pathology may start and be detectable in the GI tract in the prodromal phase of PD perhaps before affecting the brain 4, 5, 6, 7, suggesting that GI aSyn detection could be used as a predictive biomarker. According to a recent popular hypothesis, the misfolding of aSyn initially occurs in the ENS as a result of some environmental or genetic insult that then further spreads to CNS via vagal preganglionic innervation of the gut 8. This theory has been supported by *in vivo* evidence showing the progression of pathology from the periphery to the CNS following intramuscular 9 and gastric 10 injection of aSyn fibrils as well as vagotomy being associated with a decreased risk of PD 11.

It has, however, become increasingly apparent that the high sensitivity and specificity of GI aSyn detection to identify PD patients reported in the earlier studies 12, 13, 14, 15, 16 has not been sustained in subsequent studies repeatedly detecting aSyn accumulation also in the GI tract of neurologically healthy individuals 17, 18, 19, 20, 21. This was also confirmed by a recent multicentre study, which showed limited diagnostic value for detecting aSyn deposition in the GI biopsies by immunohistochemistry (IHC) 22. Noticeably, heterogeneity of antibodies, antigen retrieval methods, and sites of the GI tract assessed has produced significant variability in morphological patterns, which has confounded interpretation of what is pathological compared to nonspecific (or possibly physiological) staining.

This study was designed to tackle the limitations and issues emerging from previous studies on GI aSyn detection as a potential biomarker for PD. We detected aSyn in paraffin‐embedded GI biopsies from longitudinally followed PD patients and healthy controls (HC) participating in the Oxford *Discovery* study 23 using three different techniques: conventional IHC, our recently developed Proximity Ligation Assay for aSyn (AS‐PLA) 24 and Paraffin Embedded Tissue Blot for aSyn (AS‐PET‐blot) 25. Each technique is specific for a particular variant or conformation of aSyn. For IHC, antibodies reactive for total (T‐aSyn‐Ab) and phosphorylated (P‐aSyn‐Ab) aSyn were selected as they have been most widely used for the detection of aSyn in the GI tract making our results comparable with these previous studies 4, 14, 15, 17, 19, 20. We also used recently developed antibodies specific for the oligomeric forms of aSyn (O‐ASN‐Abs) 26, as transformation of monomeric aSyn to oligomeric conformations is increasingly recognized as the early, key pathological event in the fibrillization process of aSyn 27. With AS‐PLA 24, we targeted oligomeric aSyn and with AS‐PET‐blot fibrillar, synaptic aSyn, neither which can be detected with conventional IHC. Furthermore, we focused to describe in detail what kind of morphological staining patterns we could detect with each technique and whether we interpreted them as pathological or nonspecific. Finally, we reported each staining pattern in the GI biopsies taken both prior and after the clinical diagnosis of PD in order to see if they had predictive or diagnostic value as biomarkers.

## Methods

### Subjects

The Discovery cohort (http://opdc.medsci.ox.ac.uk) of the Oxford Parkinson's Disease Centre (OPDC) is currently one of the largest and best‐characterized longitudinal cohorts of PD subjects and HC in the world 23, 28. Full details of the protocols have been described previously 29. Briefly, validated questionnaires were used to assess and quantify a range of clinical items in this cohort including: motor function (MDS UPDRS part III); cognitive impairment (MMSE); constipation (Honolulu‐Asia Ageing Study Constipation Questionnaire); hyposmia (the 16‐stick Sniffin odour identification test); RBD (Epworth Sleepiness Scale and RBD Screening Questionnaire). All participants were systematically screened for previous biopsies of the GI tract after ethics committee approval and informed consent obtained prior to tissue request and retrieval. According to whether the biopsy had been performed before or after clinical diagnosis of PD, PD cases were classified as either ‘Prodromal PD’ or ‘Manifest PD’, respectively.

### Gastrointestinal Biopsies

Study specimens were residual tissue from biopsies or surgical resections (e.g. polyps, cancer screening) from various regions of the GI tract, including large and small intestine, stomach and oesophagus. 5 μm‐thick sections were stained with routine haematoxylin and eosin for standard histopathological evaluation by a neuropathologist (JA) to confirm the anatomical localization and quality of the specimens. Any biopsies consisting entirely or predominantly of abnormal tissue (e.g. carcinoma) were excluded.

### Immunohistochemistry

Three different antibodies reactive for aSyn were applied with optimized antigen retrieval (AR) procedures: KM51, reactive against total length of aSyn 1‐140 (Novocastra, 1:1000, AR with autoclave in citrate buffer pH 6.0 followed by 10 min incubation in 99% formic acid); pSyn#64, reactive against phosphorylated aSyn at serine 129 (WAKO, 1:10 000, AR with 5 min incubation in 20ug/ml PK); O2, reactive to fibrillary and oligomeric aSyn conformations (gift from Dr El‐Agnaf, 1:10.000, AR with autoclave in citrate buffer pH 6.0). To detect and quantify nervous tissue, two antibodies were used; Anti‐Calretinin (5A5, Novocastra, 1:200) and Anti‐Hu C/D (Thermofisher, 1:1000); with autoclave‐based, heat‐mediated AR in citrate buffer (pH 6.0) for both. For detection, Dako REAL™ EnVision™ Detection System was used with diaminobenzidine as chromogen. Assessment of aSyn immunoreactivity (absence/presence and immunostaining pattern) was carried out by two observers independently (CR, LP) without knowledge of the clinical history or diagnosis.

### Proximity ligation assay

AS‐PLA was carried out using Duolink^®^ *in situ* detection kit (Sigma) according to the manufacturer's instructions. Briefly, the conjugates were prepared using the Duolink^®^ Probemaker kit by incubating 20 μl of mouse 211 anti‐alpha‐synuclein antibody (ab80627, Abcam) with the Probemaker activated oligonucleotide (+ or ‐) and conjugation buffer overnight at room temperature (RT). The conjugates were then incubated with Probemaker stop reagent for 30 min at RT and suspended in Probemaker storage buffer and stored at +4°C. 5 μm‐thick paraffin‐embedded sections were dewaxed in xylene and rehydrated in graded ethanol series. AR was carried out with autoclave in citrate buffer pH 6.0 followed by blocking endogenous peroxidases with hydrogen peroxide for 15 min at RT. For coimmunofluorescence, sections were blocked in 10% normal goat serum, 1 M glycine TBS + 0.1% Triton and incubated for 1 h in primary antibody; Calretinin (5A5, Novocastra, 1:100), washed with TBS + 0.1% Triton and incubated for 1 h in the dark with Alexa488 (Life Technologies). Sections were washed in TBS + 0.1% Tween 20 (TBS‐T) and incubated in Duolink^®^ block solution at 37°C for 1 h, followed by the conjugates diluted in Duolink^®^ PLA probe diluent (1:100) overnight at 4°C. After washing in TBS‐T, sections were incubated with Duolink^®^ ligation solutions and ligase for 1 h at 37°C followed by Duolink^®^ amplification reagents and polymerase for 2.5 h at 37°C. For fluorescence, sections were washed in the dark and counterstained with DAPI 1:1000 and mounted with FluorSave (Millipore). For brightfield, sections were washed and incubated with Duolink^®^ detection solution for 1 hr at RT followed by Duolink^®^ substrate solution for 20 min at RT. Each step was followed by washes in TBS‐T (3 × 5 min). Sections were then counterstained with haematoxylin (Vector labs) and dehydrated in graded ethanol series and xylene, before mounting with DPX mounting reagent (Sigma).

All fluorescent images were acquired with an EVOS FL autoimaging system at ×20 magnification and automatically analysed with Cell Profiler for nuclear DAPI counting. ImageJ was used for the automatic quantification of AS‐PLA‐positive diffuse puncta and double‐labelled AS‐PLA and Calretinin‐positive cells were counted manually (NBV) blinded to the clinical diagnosis. Four random images were taken and analysed in order to provide a representative sampling of the tissue. Counts are expressed as average AS‐PLA‐positive diffuse puncta/AS‐PLA+calretinin‐positive cellular counts relative to the number of nuclei present in each imaging field.

### Paraffin embedded tissue blot

5 μm‐thick paraffin‐embedded sections were blotted onto 0.45 μm pore‐size nitrocellulose membranes (Biorad) and dried at 55°C for 24 h to improve adhesiveness of the tissue to the membrane. Sections were then dewaxed in xylene followed by rehydration in propanol series progressively diluted in distilled water (dH_2_O) (100%, 95%, 85%, 70%) and finally in dH_2_O. Sections were then dried for at least 4 h, excess membrane around the area with blotted tissue cut and sections placed into six‐well plates. Sections were incubated in TBS‐T for 30 min followed by incubation in PK (50 μg/ml) at 55⁰C for 3 h according to our optimized protocol. After PK incubation, sections were washed in TBS‐T (3 × 5 min) and incubated in denaturing buffer containing 3M Guanidine isothiocyanate followed by further washes with TBS‐T (3 × 5 min) and blocking with casein (Vector Laboratories) for 30 min. Samples were then incubated overnight at RT with primary antibody; LB509 (Millipore, 1:10 000). After washing with casein (3 × 5 min), sections were incubated with secondary anti‐mouse biotinylated antibody (1:1000) for 1 h, washed again with casein (3 × 5 min), and then incubated with avidin‐biotin complex (Vectastain ABC‐AmP, Vector Laboratories) for 25 min. After further washes with casein (3 × 5 min), labelling was developed using alkaline phosphatase BCIP/NBT solution (Vector Laboratories). All incubations were carried out on a shaking table (except for PK incubation in the oven) with 5 ml solution/well. Finally, sections were abundantly rinsed in distilled water. After air‐drying, sections were briefly dipped in xylene and mounted using DPX mounting reagent (Sigma), which enabled visualization with a normal light microscope. Digital images of slides at ×40 magnification were obtained using Aperio Scanscope and each blot was examined by two observers (IP, CR) without knowledge of the clinical history or diagnosis.

### Statistical analysis

Univariate comparisons between two groups (PD vs HC) were made using the t‐test or Fisher's exact test/Chi‐square. For comparisons between three groups (Prodromal PD, Manifest PD, and HC), we used one‐way ANOVA or Fisher's exact test/Chi‐square. We used t‐test, ANOVA and Pearson or nonparametric Mann–Whitney, Kruskal–Wallis and Spearman to evaluate relationship and correlations between clinical, demographic and pathological variables of interest. The measure of agreement between categorical assessments of the two techniques was estimated applying the Cohen kappa. Receiver Operator Characteristic (ROC) curves were constructed to evaluate the ability of pathology to predict the clinical status.

## Results

### Clinical and demographic characteristics

Demographic and clinical information is given in **Table** 1. In the overall cohort, the mean age at biopsy was 65.3 ± 9.9 years. Thirty‐one PD subjects were classified as ‘Prodromal PD’, and in this group the mean time from biopsy to PD diagnosis was 5.8 ± 3.8 years. In the 20 ‘Manifest PD’ patients, the mean time from PD diagnosis to GI biopsy was 1.6 ± 1.4 years. The ‘Manifest PD’ group was significantly older at the time of biopsy compared to both ‘Prodromal PD’ cases (*P* = 0.007) and HC (*P* = 0.015). The mean age at PD diagnosis was 69.1 ± 8.2 years and did not differ between the ‘Prodromal’ and ‘Manifest’ PD groups. The earliest GI biopsy was performed 17 years prior to the diagnosis of PD, while the longest duration of disease at the time of GI biopsy was 5 years.

**Table 1:** Demographic and clinical characteristics across the clinical groups

	Prodromal PD	Manifest PD	Healthy Controls	*P*‐value
*N*	31	20	21	–
Males	16 (52)	13 (65)	7(33)	0.140
Age at biopsy	63 (43–83)	71 (57–85)	62 (39‐86)	**0.014**
Age at diagnosis	69 (56–87)	69 (55–83)	–	0.342
Biopsy – Diagnosis	6 (1–17)	−2 (−5–0)	–	**<0.001**
UPDRS III	26 (6–41)	35 (14–68)	3 (0–10)	**<0.001**
MMSE	28 (23–30)	28 (20–30)	29 (24–30)	0.181
MOCA	24 (16–30)	24 (17–29)	27 (21–30)	**0.036**
Constipation	17 (57)	4 (22)	7 (33)	**0.045**
RBD	16 (52)	8 (42)	4 (20)	0.077
Hyposmia	21 (68)	13 (72)	3 (15)	**<0.001**
Probability PD	90	90	–	0.991

Values are presented as number of cases, with range or % in parenthesis, as appropriate. Groups were compared with one‐way ANOVA, independent samples T‐test or Chi‐Square test, as appropriate. Numbers in bold represent significant differences between groups.

The UPDRS motor score was significantly higher in the ‘Manifest PD’ compared to ‘Prodromal PD’ (*P* = 0.005) and in both PD groups compared to HC (*P* < 0.001). The MOCA scores were significantly lower in both ‘Manifest PD’ (*P* = 0.017) and ‘Prodromal PD’ (*P* < 0.001) compared to HC but did not differ between two PD groups. There were no significant differences amongst the three groups in MMSE. Prevalence of constipation was significantly different (*P* = 0.045); being highest in the ‘Prodromal PD’ (present in 57%), followed by HC (present in 33%) and ‘Manifest PD’ (present in 22%). Prevalence of hyposmia was also statistically different (*P* < 0.001); seen equally in ‘Manifest PD’ (72%) and ‘Prodromal PD’ (68%), whereas only 15% of HC suffered from a loss of smell. There were no differences in prevalence of REM sleep behaviour disorder (RBD) and probability of PD.

### Gastrointestinal tissue biopsies

We retrieved a total of 113 tissue blocks from 51 PD patients and 50 blocks from 21 HC, from ~200 Discovery participants declaring a previous GI biopsy. Recall bias or colonoscopy being done without any sampling explained the missing biopsies. Staining was performed on tissue sections from one or more GI tract regions from 72 subjects (51 PD and 21 HC). A single GI region was examined in 53 cases (38 PD and 15 HC), two regions in 13 cases (9 PD and 4 HC) and three regions in six cases (4 PD and 2 HC). Large bowel was studied in 101 blocks (35 PD and 12 HC), small bowel in 34 (19 PD and 10 HC), stomach in 12 (7 PD and 5 HC), and oesophagus in 11 (7 PD and 2 HC).

### Immunohistochemistry

#### Neuronal staining

*S*emiquantitative assessment of the intensity of staining (*i.e*. absent to intense) with the neuronal marker (calretinin/Hu) was carried out to estimate the density of nervous tissue, including mucosal nerve fibres and submucosal ganglionic neurones (Table 2, Figure 2 **E–G**). There were no significant differences in the neuronal score of GI biopsies or in the prevalence of submucosal ganglionic neurones between PD and HC.

**Table 2:** Baseline histological characteristics of the GI biopsies

	PD	HC	*P*‐value
Semiquantitative neuronal score:			
0 (none)	24%	22%	0.451
1 (some)	25%	17%	
2 (moderate)	37%	36%	
3 (severe)	14%	25%	
Submucosa present (Y/N)	47%	61%	0.143
Submucosal ganglia present (Y/N)	28%	35%	0.449
Number of submucosal ganglia (Total)	64	43	0.129
Myenteric plexus present (Y/N)	8%	11%	0.525

#### aSyn staining

**Figure 1:**
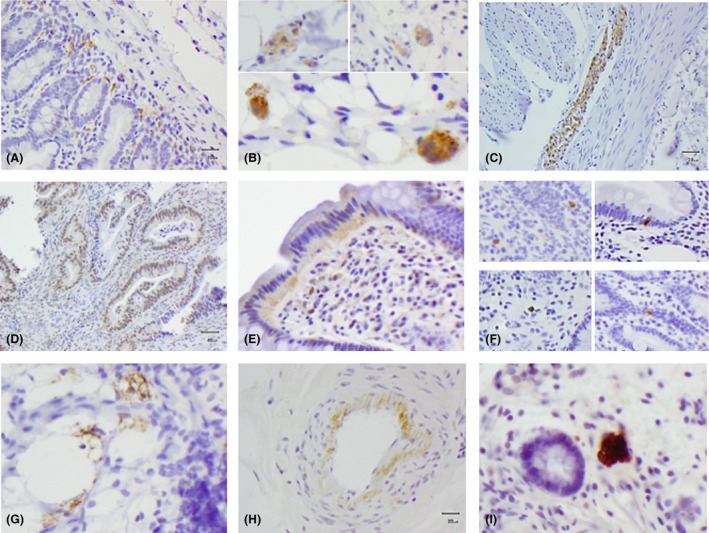
Photomicrographs of aSyn staining patterns with immunohistochemistry: Neuritic (**A**); Ganglionic (diffuse/granular) (**B**); Myenteric (**C**); Epithelial (**D, E**); Cellular (**F**); Lacy‐Granular (**G**); Perivascular (**H**); Macrophages (**I**).

**Figure 2:**
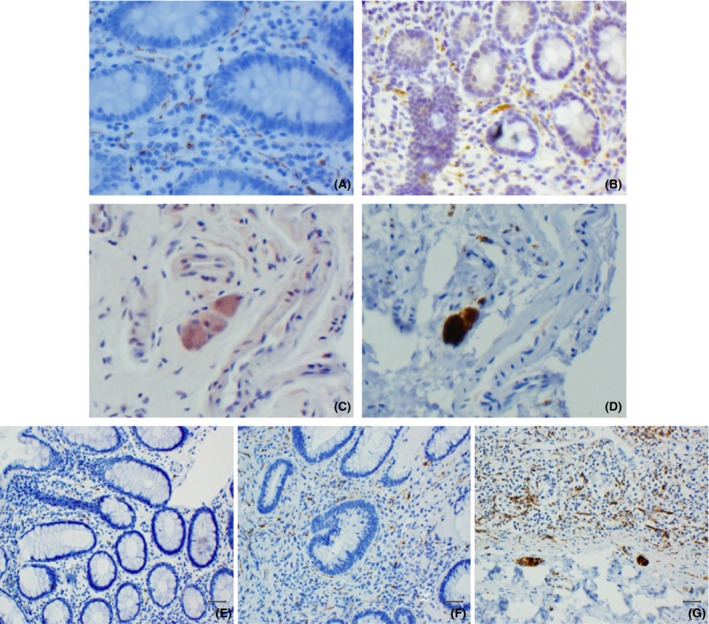
aSyn‐reactive antibodies revealed neuritic‐like pattern in the mucosa (**A**) and diffuse, intraneuronal staining of some submucosal ganglionic neurones (**C**) which resembled the morphological pattern of staining obtained with the neuronal marker (calretinin) in consecutive sections (**B, D**). Semiquantitative grading of neuronal marker, calretinin. 1 = Low density of nerve fibres; no ganglionic cells (**E**); 2 = Low density of nerve fibres and ≥ 1 ganglionic cell, or moderate‐high density of nerve fibres without ganglionic cells (**F**); 3 = Moderate‐high density of nerve fibres and ≥ 1 ganglionic cell (**G**). Magnification ×100 **A, B, F** and ×200 in **C, D, E, G**.

All cases had at least one tissue block stained with each of the three antibodies reactive for aSyn. Overall positive staining for aSyn IHC was found in GI tissue sections of 31/51 (61%) PD patients (21 Prodromal and 10 Manifest) and 13/21 (62%) HC. Several different staining patterns were observed (Figure 1). Two of these were suggestive of neuronal localization of aSyn: neuritic‐like distribution in the mucosa (*neuritic* staining, Figures 1 **A**, 2 **A**) and diffuse/granular pattern seen inside the submucosal ganglionic cells (*ganglionic* staining, Figures 1 **B**, 2 **C**) that often colocalized with our neuronal marker in adjacent sections (Figures 2 **B,D**). Diffuse, dense staining in the myenteric plexus was also seen in the few cases where this was present in the biopsy (Figure 1 **C**). Other types of staining observed in a significant proportion of cases, on the other hand, were suggestive of a non‐neuronal localization. The most prevalent of this type of staining was found in the cytoplasm of epithelial cells lining the gastric glands of the mucosa, (*epithelial* staining, Figure 1 **D,E**) or in other cellular components of the mucosa (*cellular* staining, Figure 1 **F**). In a small number of cases, further staining patterns were also observed, including *lacy‐granular* staining (Figure 1 **G**), *perivascular* staining (Figure 1 **H**) and aSyn‐positive *macrophages* (Figure 1 **I**). These were, however, very rare and judged to be nonspecific staining (not included in further analysis).

Different antibodies showed varying sensitivities towards different staining patterns observed. The staining was most prevalent with the O‐aSyn‐Ab, which detected any neuronal (neuritic/ganglionic) staining in 13 of 31 (42%) Prodromal PD patients; 7 of 20 (35%) Manifest PD patients and 5 of 21 (24%) HC. The P‐aSyn‐Ab revealed neuronal staining in 4 of 31 (13%) Prodromal PD patients; 3 of 20 (15%) Manifest PD patients and 5 of 21 HC (24%), whereas the T‐aSyn‐Ab revealed no positive neuronal staining in any of the examined cases. Interestingly, the T‐aSyn‐Ab showed aSyn staining in only three subjects, with cellular‐like staining in the mucosa in 2 PD patients and an epithelial pattern in one HC.

When we compared the prevalence of various staining patterns between PD and HC and between Prodromal PD, Manifest PD and HC groups using any of our three antibodies, there were no statistically significant differences (Table 3), even when the four HC subjects with RBD were removed from analysis. Grouped neuronal (*neuritic* or *ganglionic*) or non‐neuronal (*epithelial* or *cellular*) staining also did not differ between the groups. ROC curve analysis showed that neither the neuronal or non‐neuronal staining were accurate predictors of the clinical status (AUC = 0.578 for neuronal, AUC = 0.501 for non‐neuronal staining). Furthermore, none of the clinical variables (UPDRS, MMSE, MOCA, constipation, RBD, hyposmia) were statistically different in the group with GI aSyn neuronal staining compared to the group without. When prevalence of aSyn staining was compared by presence or absence of submucosa in the tissue sample, there was a significantly higher prevalence of aSyn staining in the group with submucosa, which is not surprising. However, when we included only samples with submucosa available, there was no significant difference in prevalence of aSyn staining between PD and HC groups. There was no correlation between age at biopsy and prevalence of aSyn staining.

**Table 3:** Prevalence of staining patterns across the clinical groups

	PD	Prodromal PD	Manifest PD	HC	PD vs. HC (*P*‐value)	Prodromal PD vs. Manifest PD vs. HC (*P*‐value)
Number	51	31	20	21		
aSyn IHC Neuronal						
Neuritic (mucosal)	18 (35)	10 (32)	8 (40)	4 (19)	0.174	0.334
Ganglionic (submucosal)	14 (27)	8 (26)	6 (30)	7 (33)	0.499	0.838
Any neuronal (neuritic or ganglionic)	25 (49)	16 (52)	9 (45)	7 (33)	0.441	0.428
aSyn IHC Non‐Neuronal						
Epithelial	18 (35)	13 (42)	5 (25)	10 (48)	0.330	0.299
Cellular	9 (18)	4 (13)	5 (25)	2 (10)	0.384	0.344
AS‐PLA^a^						
Cellular	0.02 ± 0.03	0.02 ± 0.04	0.02 ± 0.02	0.02 ± 0.02	0.737	0.716
Diffuse	0.69 ± 0.49	0.65 ± 0.51	0.74 ± 0.48	0.76 ± 0.50	0.597	0.662
AS‐PET‐blot						
aSyn‐localized	9 (18)	5 (16)	4 (20)	8 (38)	0.063	0.170
Pericrypt	30 (59)	19 (61)	11 (55)	12 (57)	0.217	0.423

Values are presented as numbers and (% within clinical subgroup) except AS‐PLA Diffuse and Cellular variables, which are presented as mean score ± SD.

a Group comparison carried out with nonparametric Mann–Whitney or Kruskal–Wallis test.

Analysis of IHC staining patterns by GI region did reveal significant differences in their distribution (*P* < 0.001); there was significantly more neuronal aSyn staining in the small bowel (47%) followed by oesophagus (27%) compared to stomach and large bowel (both 13%). The prevalence and intensity of non‐neuronal staining was much greater in the distal GI tract (*i.e*. small and large bowel) and was not found in the oesophagus or stomach (Table 4).

**Table 4:** Prevalence of different staining types across different GI regions

	Oesophagus	Stomach	Small Bowel	Large Bowel	*P*‐value
aSyn IHC Neuronal (*n*)	11	16	34	102	
Neuritic (mucosal)	3 (27)	2 (13)	11 (32)	2 (2)	**<0.001**
Ganglionic (submucosal)	0	1 (6)	11 (32)	11 (11)	**0.004**
Any neuronal (neuritic or ganglionic)	3 (27)	2 (13)	16 (47)	13 (13)	**<0.001**
aSyn IHC Non‐Neuronal (*n*)	11	16	34	102	
Epithelial	0	0	14 (41)	20 (20)	**0.001**
Cellular	0	0	3 (9)	11 (11)	0.391
AS‐PLA^a^ (*n*)	5	9	27	63	
Cellular	0.04 ± 0.08	0.01 ± 0.01	0.02 ± 0.02	0.02 ± 0.02	**0.020**
Diffuse	0.86 ± 0.53	0.89 ± 0.49	0.58 ± 0.39	0.65 ± 0.54	0.239
AS‐PET‐blot (*n*)	5	5	23	56	
aSyn‐localized	0	0	7 (30)	10 (18)	0.228
Pericrypt	2 (40)	2 (40)	10 (43)	34 (61)	0.479

Values are presented as number of positive blocks (% of all the block stained of the GI region).

a Group comparison carried out with nonparametric Kruskal–Wallis test. Numbers in bold represent significant differences between groups.

### Proximity ligation assay

**Figure 3:**
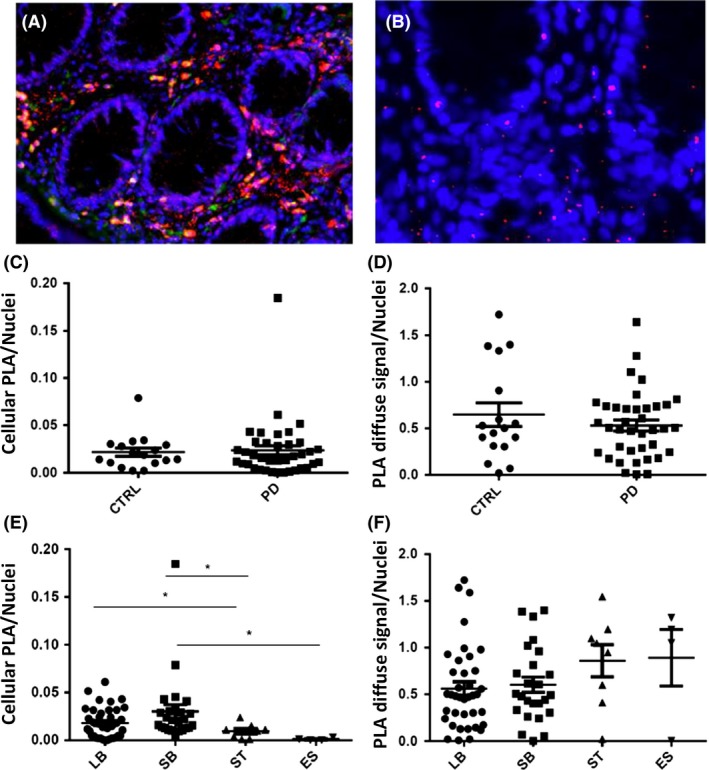
AS‐PLA (red) – calretinin (green) double immunofluorescence detected two types of staining (**A**) cellular signal *i.e*. cellular ‐like formations in the mucosa partly costained with the neuronal marker calretinin; (**B**) diffuse signal *i.e*. a dot‐like pattern detected throughout the section without any specific anatomic localization. Magnification ×100 in **A**, ×200 in **B**. CTRL: Control; ES: Oesophagus; LB: Large Bowel; PD: Parkinson's Disease; SB: Small Bowel; ST: Stomach.

104 blocks were stained from 58 individuals (40 PD and 18 HC) for AS‐PLA. Immunofluorescent AS‐PLA was optimized in order to achieve a comparable signal to brightfield AS‐PLA followed by double immunolabelling with the neuronal marker calretinin. Two types of staining were detected with AS‐PLA‐calretinin double immunofluorescence: (i) cellular‐like formations in the mucosa, which partly costained with the neuronal marker calretinin (i.e. *cellular* signal) and (ii) a dot‐like pattern detected throughout the section, with no specific anatomic localization (i.e. *diffuse* signal) (Figure 3). AS‐PLA cellular signal was statistically higher in the group with high neuronal score compared to low (*P* = 0.008) and correlated significantly with the number of ganglia/section (*P* = 0.026). However, the level of agreement between neuronal aSyn IHC staining and cellular AS‐PLA signal (which we divided into low and high by a mean value of 0.02) was poor (*P* = 0.095, κ = −0.04), with 41% (43/104) of all blocks showing a contradictory result in terms of aSyn positivity.

In 104 blocks, the mean score for the cellular and diffuse AS‐PLA signals were 0.02 ± 0.02 and 0.66 ± 0.50, respectively, but there were no significant differences in either signal between any of the study groups (Table 3, Figure 3C‐D). Furthermore, the two different types of AS‐PLA staining did not correlate with each other. ROC curve analysis showed that neither signal was a reliable predictor of the clinical status (AUC = 0.737 for cellular and AUC = 0.597 for diffuse AS‐PLA signal). Neither AS‐PLA signal correlated with UPDRS motor score or MMSE but the cellular AS‐PLA signal (not diffuse) correlated positively with MOCA (*P* = 0.030, *r*_s_ = 0.288). AS‐PLA cellular/diffuse signals were not significantly different in subjects who were constipated or had hyposmia compared to subjects who did not present with these symptoms. However, the mean diffuse AS‐PLA signal was found statistically higher (*P* = 0.020) in subjects without RBD (0.84 ± 0.51) than with RBD (0.54 ± 0.42).

The cellular AS‐PLA signal was statistically higher in the large (*P* = 0.038) and small bowel (*P* = 0.012) compared to stomach, in small bowel compared to oesophagus (*P* = 0.024) (Figure 3E). The diffuse AS‐PLA signal showed no differences with regard to GI tract distribution (Table 4, Figure 3F).

### Paraffin‐embedded tissue blot

**Figure 4:**
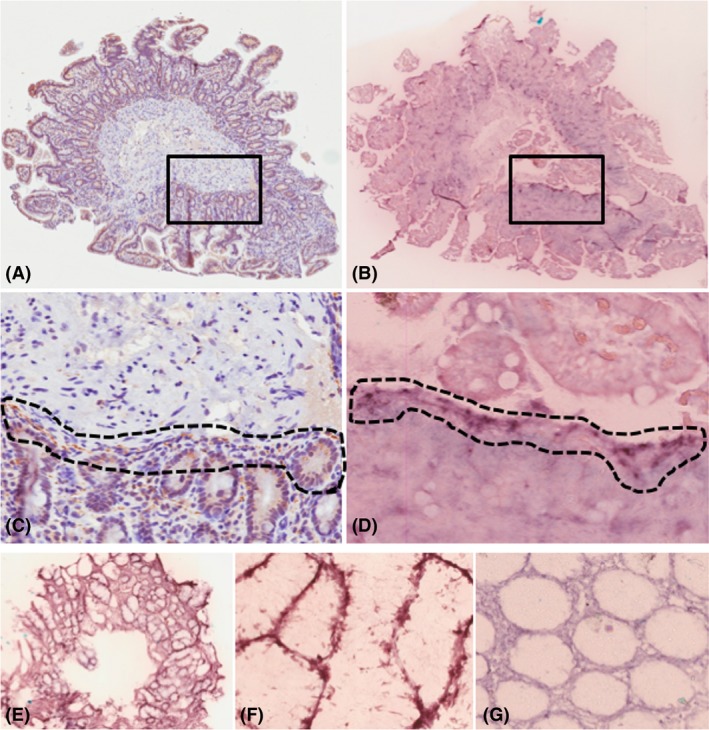
Some aSyn‐positive PET‐blot staining (**B, D**) colocalized with staining observed with immunohistochemistry (**A, C**). Pericrypt AS‐PET‐blot signal in colonic tissue of Parkinson's disease patient at low ×40 (**E**) and high ×200 (**F**) magnification. Absence of a similar signal in colonic tissue of another PD case (**G**). Magnification ×10 in **A**,** B** 100 × in **C**,** D**. Antibodies: IHC ‐ O2; PET‐Blot – LB509.

89 blocks were stained with the AS‐PET‐blot technique from 51 PD patients and 21 HC, so that at least one tissue block was stained from each case. This method was first optimized using brain tissue and revealed numerous aSyn micro‐aggregates in the striatum of a PD patient that were not detected with conventional IHC. The most frequently occurring signal we observed with this technique in GI biopsy tissue was present in more than half (55%) of the examined blocks and consisted of staining along the borders of the mucosal glands or crypts (*pericrypt* staining, Figure 4 **E–F**). Another type of staining occasionally colocalizing with aSyn deposition detected with IHC (*aSyn‐localized* staining, Figure 4 **A–D**) was observed in 17 (19%) out of 89 examined blocks. Despite the partial colocalization, the level of agreement between neuronal aSyn IHC staining and PET‐blot aSyn‐localized signal was poor (*P* = 0.709, κ = 0.04), with 34% (30/89) of all blocks showing a contradictory result in terms of aSyn positivity. Furthermore, no statistical differences in either pericrypt or aSyn‐localized staining patterns were found between the study groups (Table 3). ROC curve analysis showed that aSyn‐localized PET‐blot signal was not a reliable predictor of the clinical status (AUC = 0.398). In contrast, the UPDRS score was significantly lower (*P* = 0.020) among those cases that showed the aSyn‐localized staining (13 ± 12) compared to ones without (24 ± 17). There were no differences in MMSE score, but MOCA was also significantly higher (*P* = 0.020) among the cases with aSyn‐localized staining (27 ± 2) compared to ones without (25 ± 3). The prevalence of aSyn‐localized signal was not significantly different in relation to constipation, RBD or hyposmia and showed no difference with regard to GI tract distribution.

## Discussion

In this study, we approached detection of aSyn in the ENS as a potential biomarker for PD, with the goal of increasing sensitivity and specificity and gaining a better understanding of early pathogenic mechanisms in the PD GI tract. IHC, AS‐PLA and AS‐PET‐blot were applied, each targeting a specific conformational/post‐translational variant of aSyn, namely (i) total (monomeric), (ii) phosphorylated, (iii) oligomeric and (iv) synaptic, fibrillar aSyn. We described the morphological staining patterns detected with each technique and their possible interpretation as pathological.

With IHC, positive aSyn staining was found in 61% of PD patients and in 62% of HC. Putatively pathological neuronal aSyn staining was detected in a smaller proportion of subjects but again with no significant difference between PD (49%) and HC (33%). Thus, similarly to other studies examining sizeable archived biopsy samples 4, 6, the sensitivity of neuronal aSyn staining was poor. Furthermore, unlike others 14, we found no relationship between neuronal aSyn staining and any of our detailed clinical features (motor and cognitive scores, constipation, RBD, hyposmia). Interestingly, neuronal aSyn pathology was recently found in colonic biopsies of 4 out of 17 (24%) patients with RBD 7, who carry an 80% risk of developing synucleinopathy 30. The earliest time point we detected any neuronal aSyn pathology was 10 years before the development of clinically manifesting PD, but no difference was seen between ‘Prodromal PD’ (52%) vs. ‘Manifest PD’ (45%) groups, suggesting that there is no cumulative effect over time. With regards to clinico‐pathological correlation, it must be noted that there was significant variability in the time lag between biopsy date and the time of clinical characterization, thus this correlation has potentially limited relevance. In a small number of cases, more than one GI region was studied. While this was not systematically assessed in this study, comparing aSyn staining patterns between different GI regions of the same subject could address the issue of differential susceptibility to aSyn accumulation and transmission.

The overall specificity in our study was 67% (i.e. 7 of our 21 HC showed neuronal aSyn pathology). In previous studies, the specificity has ranged extensively from near 100% 4, 13, 14, 15, 16 to 0% 17, 18, 19. Varying sensitivity and specificity of aSyn antibodies recognizing different epitopes is a well‐described phenomenon in the brain 31, 32 but was recently also demonstrated in the GI tract 33. Harmonizing terminology used to describe different morphological patterns of aSyn immunoreactivity seen in the GI tract and their biological interpretation (pathological vs. nonspecific/physiological) is also emerging 33, 34. Similar to a recent consensus paper 34, we observed granular staining in the mucosa/lamina propria, which we termed as *neuritic,* since it is suggestive of localization in the peripheral nerve endings as demonstrated by staining with the neuronal marker (i.e. calretinin). In addition, roughly one‐third of our sections contained submucosa, where aSyn staining was observed in the ganglionic cells with a diffuse or granular pattern (defined as *ganglionic*) that also overlapped with the neuronal staining. Thus, both of these staining patterns (i.e. *neuritic* and *ganglionic*) were considered to be specifically neuronal and putatively pathological. Different antibodies showed drastically different sensitivities (and specificities) for the neuronal pattern of staining, with the highest sensitivity being achieved with the O‐aSyn‐Ab, with a positive signal in 39% of PD vs. 24% HC, followed by the P‐aSyn‐Ab with 14% of PD vs. 24% HC, and finally T‐aSyn‐Ab that surprisingly, and in contrast with other studies 4, 15, 19, 20, 33 showed no neuronal staining in any of the examined cases. We found no difference in specificity whether we used O/P‐aSyn‐Ab. Although phosphorylation is generally considered a marker of choice to delineate pathological aggregates from normal, native aSyn accumulation 35, using the P‐aSyn‐Ab, Bottner *et al*. 17 and Visanji *et al*. 19 detected neuronal aSyn pathology in all of their HC. In another study, colonic biopsies from PD and HC showed no difference in phosphorylated aSyn expression levels measured by Western blotting 36. These findings emphasize the need for a better understanding of aSyn phosphorylation in PD pathogenesis both in the brain and in the GI tract 37, 38. Improved quantification of aSyn staining could also benefit sensitivity and specificity. To date, studies have mostly used qualitative assessment of sections to define pathology, and only a few studies have attempted quantification with semiquantitative grading scales to measure the severity of staining 14, 20, 39. Virtual microscopy and whole‐slide imaging could allow a more robust quantitative analysis of enteric aSyn and potentially facilitate definition of reliable cut‐off scores to distinguish PD patients from controls.

*In vivo* endoscopic biopsies are willingly kept superficial for safety reasons (i.e. to prevent bleeding), and consequently these specimens usually reveal dot/thread‐like aSyn deposits in the peripheral nerve endings in the mucosa but rarely show ganglionic perikaryal inclusions. The empirical categorization as pathological or not is based on morphological features and double‐labelling with neuronal markers, but the encountered aSyn staining patterns are not always easily traceable to a definite anatomic localization and at times are suggestive of nonspecific, possibly even artefactual staining. Indeed, in addition to neurons, aSyn is physiologically expressed in hematopoietic 40, endothelial and neuroendocrine cells 41 and in muscle fibres 42. We found several cases with staining of *epithelial* cells of gastric glands that was considered to be a nondisease‐related cross‐reaction to some secretory protein or enzymes of the gastric mucosa 33. *Cellular* elements detected, on the other hand, could represent mucosal macrophages ingesting pathological aSyn but their role in PD pathogenesis remains to be established 18. Therefore, the detection of non‐neuronal aSyn does not necessarily indicate a disease state. Conversely, we cannot currently rule out the potential pathogenic role of non‐neuronal aSyn either. Indeed, plasma cells, neuroendocrine and smooth muscle cells, which are highly represented in the GI tract (as opposed to the CNS) and located in close proximity to neuronal elements, could represent possible sources of aSyn be taken up by neurones, and further studies assessing transport of aSyn between neuronal and non‐neuronal cells would be informative 3.

The PLA was developed for *in situ* detection of interactions between different, functionally related proteins through generation of a chromogenic or fluorescent signal following DNA circularization and amplification 43. This technique was recently adapted by our group to detect oligomeric forms of aSyn resulting from interaction of two or more aSyn monomers 24. This is the first study to have applied the AS‐PLA for the detection of aSyn oligomers in the GI tract, and we hypothesized that we could detect these pathological aSyn conformations early, possibly at a prodromal PD stage. Of the two types of AS‐PLA signal that were observed, the *cellular* staining positively correlated with neuronal score and number of ganglionic neurones, which supports the idea that this staining was localized mainly to neuronal elements in the mucosa and submucosa. The *diffuse* staining pattern we observed in the GI tract was reminiscent of what is seen in the neuropil of the cortex of PD patients that may represent synaptic distribution, but this needs to be examined further. While AS‐PLA did detect oligomeric aSyn in the majority of sections stained, there was no distinctive pattern or quantitative threshold that could distinguish between PD patients (Prodromal or Manifest PD groups) and HC. Furthermore, the *cellular* AS‐PLA signal was actually found to be higher in the patients that performed better in their cognitive testing and the *diffus*e AS‐PLA signal lower in those patients with RBD compared to those without. Thus, clearly, the role of oligomeric forms of aSyn in the GI tract needs further elucidation, but it could be that these conformations are rather transient and their quantification in a single time point may not be a meaningful approach as a biomarker.

Finally, considering that aSyn aggregation likely begins in the axon terminals (i.e. peripheral nerve endings), we hypothesized that PET‐blot would be ideal to detect this early pathology in the GI tract. This protein detection technique was originally developed to detect pathological prion protein 44 and is based on the incubation of paraffin‐embedded tissue with proteinase K (PK), a 28.9 KDa serine protease with broad specificity that digests proteins through cleavage of peptide bonds. Importantly, the monomeric, physiological form of aSyn is digested with PK treatment, whereas the aggregates of misfolded and insoluble aSyn resist digestion 45, 46, increasing the visualization of smaller aSyn micro‐aggregates, that most likely represent synaptic pathology 25, 47. Despite successful optimization of AS‐PET‐blot in the PD brain to reveal striatal aSyn micro‐aggregates undetectable by conventional IHC, we were unable to obtain a similar, specific signal in the GI tissue. This could be due to several reasons, such as that GI tissue has a different fat/soft/connective tissue composition compared to CNS, and this may at least partly explain the higher propensity of GI biopsy tissue to be morphologically damaged by the PK treatment. Our assessment of optimal PK concentrations, incubation temperatures and durations in the GI tract resulted in ‘softer’ conditions than those used in the brain, which improved morphological quality of the stained sections but may have also interfered with the PET‐blot's ability to reveal the synaptic aSyn microaggregates. It is also possible that aSyn conformations may differ between GI tract and brain, with the former hosting a smaller proportion of fibrillary forms of aSyn. Interestingly, some studies examining biochemical characteristics of aSyn between the ENS and CNS have shown that native aSyn occurs primarily as a monomer in enteric neurones 22, whereas in the brain dimeric and tetrameric species of aSyn are also observed 48. These differences in aSyn conformations may explain the discrepancy in our results with AS‐PET‐blot between ENS and CNS. Similar to an earlier study by Visanji *et al*. 19, we did observe coarser accumulations of aSyn without specific morphological localization with AS‐PET‐blot that partially colocalized with aSyn IHC in adjacent sections; however, these did not allow distinction between PD and HC. In addition, no correlation with severity of clinical symptoms was found, and in contrast, the group with aSyn‐localized PET‐blot signal performed better in both motor and cognitive testing than the group without these changes in the GI tract.

## Conclusions

In this study, for the first time, the GI tract was targeted by three different methods, each specific for alternative conformations of aSyn, to increase specificity and sensitivity of detecting pathological GI aSyn as a diagnostic biomarker for PD. However, although we detected specific staining patterns with each method, no single technique or staining pattern was able to reliably distinguish PD patients (Prodromal or Manifest PD) from HC. None of the pathological staining patterns correlated with severity of clinical symptoms; in contrast, some were more abundant in those patients who performed better with their motor or cognitive tests. Thus, our detailed description of aSyn detection in GI biopsy tissue from a large, clinically well‐characterized cohort does not support any predictive or diagnostic value for this approach. More sensitive methods such as our recently developed aSyn real‐time quaking induced conversion (AS‐RT‐QuIC) assay 49 may prove more helpful than tissue‐based detection techniques for different aSyn conformations.

## Author Contributions

MH and LP obtained the funding for this study; CR, MH, LP designed the study; CR and MH provided all the clinical data; CR collected the GI tract samples and performed all the IHC experiments and analysis; NBV and JA performed the AS‐PLA experiments and analysis; IP and DR performed the AS‐PET‐blot experiments and analysis; JA provided the standard histopathological evaluation of the GI biopsies; CR and LP prepared the manuscript and all authors revised the manuscript for the intellectual content.

## Disclosure

All authors declare no conflict of interest.
